# Prospects for microwave plasma synthesized N-graphene in secondary electron emission mitigation applications

**DOI:** 10.1038/s41598-020-69844-9

**Published:** 2020-08-03

**Authors:** N. Bundaleska, A. Dias, N. Bundaleski, E. Felizardo, J. Henriques, D. Tsyganov, M. Abrashev, E. Valcheva, J. Kissovski, A. M. Ferraria, A. M. Botelho do Rego, A. Almeida, J. Zavašnik, U. Cvelbar, O. M. N. D. Teodoro, Th. Strunskus, E. Tatarova

**Affiliations:** 1 Instituto de Plasmas E Fusão Nuclear, Instituto Superior Técnico, Universidade de Lisboa, 1049 Lisbon, Portugal; 2 CEFITEC, Departamento de Física, Faculdade de Ciências E Tecnologia, Universidade Nova de Lisboa, 2829-516 Lisbon, Portugal; 3 Faculty of Physics, Sofia University, 1164 Sofia, Bulgaria; 4 BSIRG, iBB, DEQ, Instituto Superior Técnico, Universidade de Lisboa, 1049-001 Lisbon, Portugal; 5 Centre of Physics and Engineering of Advanced Materiais, Instituto Superior Técnico, Universidade de Lisboa, 1049-001 Lisbon, Portugal; 6 Department of Gaseous Electronics F6, Jozef Stefan Institute, 1000 Ljubljana, Slovenia; 7 Institute for Materials Science, Christian Albrechts Universitaet Zu Kiel, Kiel, Germany

**Keywords:** Materials science, Physics

## Abstract

The ability to change the secondary electron emission properties of nitrogen-doped graphene (N-graphene) has been demonstrated. To this end, a novel microwave plasma-enabled scalable route for continuous and controllable fabrication of free-standing N-graphene sheets was developed. High-quality N-graphene with prescribed structural qualities was produced at a rate of 0.5 mg/min by tailoring the high energy density plasma environment. Up to 8% of nitrogen doping levels were achieved while keeping the oxygen content at residual amounts (~ 1%). The synthesis is accomplished via a single step, at atmospheric conditions, using ethanol/methane and ammonia/methylamine as carbon and nitrogen precursors. The type and level of doping is affected by the position where the N-precursor is injected in the plasma environment and by the type of precursors used. Importantly, N atoms incorporated predominantly in pyridinic/pyrrolic functional groups alter the performance of the collective electronic oscillations, i.e. plasmons, of graphene. For the first time it has been demonstrated that the synergistic effect between the electronic structure changes and the reduction of graphene π-plasmons caused by N doping, along with the peculiar “crumpled” morphology, leads to sub-unitary (< 1) secondary electron yields. N-graphene can be considered as a prospective low secondary electron emission and plasmonic material.

## Introduction

The development of low Secondary Electron Emission (SEE) materials is of great importance for modern technologies, overarching from space applications (e.g. telecommunication satellites) to particle accelerators. For instance, a significant potential difference between the dielectric and conductive part of satellites can arise due to a difference in their secondary electron yields (SEYs) induced by cosmic rays, igniting discharges that can disrupt normal operation or even destroy the equipment. Additionally, in the presence of an alternating electric field and under resonant conditions, secondary electrons can accelerate to high enough velocities to strike the material and free additional electrons, which can then be accelerated and so forth, leading to an exponential increase of the number of free electrons, a process known as multipacting^1–14^. This phenomenon is responsible for the formation of electron clouds (e-clouds) in particle accelerators, which affect particle beam trajectories and introduce beam instabilities. In addition, electron bombardment of the tube walls introduces pressure rise and additional heat loads of these cryogenic systems. All this makes e-cloud effect the major limitation of beam luminosity of modern particle accelerators. Similarly, e-cloud formed in microwave generators of satellite communication devices affects electron trajectories and introduces noise in the communication systems.

Since SEE occurs in the topmost 5 nm layer of the material being struck by the primary electrons, the techniques usually employed to mitigate multipacting rely on surface modification by deposition a thin film of a material with lower SEY^15^. SEE is a surface process that often is not well determined because it depends on the type of the material but also on surface finish, i.e., surface contaminants and morphology. Equally important to have surfaces with low SEY is assuring that these are chemically stable, i.e., inert, as well as conductive. In principle, the potential for electron avalanches formation is avoided by using materials with SEY < 1, but in some applications the threshold can be higher.

Altering the surface composition in order to reduce the SEY has been extensively studied. Carbon-based thin films, in particular of the amorphous kind, are routinely used for this purpose in particle accelerators due to their relatively low SEY (0.95–1.05). By applying an amorphous carbon thin film to a Stainless-Steel surface using DC Magnetron Sputtering, the SEY can be reduced from 2.4 to about 1 while leaving the bulk of the material and its properties virtually unchanged^16^. Recent investigations have demonstrated that SEY can be further reduced using a graphene coating of the surface^17,18^. Potential advantage of this material in respect to amorphous carbon is its superior chemical inertness. Sian et al. have reported a maximum SEY of 1.4 in Stainless Steel samples coated with graphene using electrophoretic deposition (EPD), down from the 2.4 for the uncoated substrate^19^. Jie Wang et al. have shown that depositing 6–8 layers of graphene in a copper substrate by CVD lowers the maximum SEY to 1.25^20^. However, considering practical applications, depositing previously synthesized graphene powder is highly relevant and advantageous as compared of direct growing graphene on the substrates. Sub-unitary values of SEY were reported by Montero et al., in aluminium surfaces coated with graphene nanoplatelets, with significant improvements being obtained by simple change of substrate roughness^21^. Although, the lack of data regarding essential characteristics of the used graphene (e.g. sp^2^/sp^3^ carbons ratio, oxygen content etc.) as well as surface finish makes difficult to assess contribution of graphene to the low SEY values reported in their work.

The search for low SEY materials to mitigate e-cloud effect has become an important technological demand. New materials with better performance should, ideally, be produced via large-scale, cost-effective, and environmentally clean technologies. In this respect, graphene could become a prime low SEY material candidate due to its electronic structure, chemical inertness^16,22^ and different means of its coating on metallic surfaces. However, in spite of significant development of the graphene market (there are about 250 companies worldwide producing graphene and its derivatives in different forms) massive application of this material is not yet achieved in any area. It has been recently argued by Kauling and co-authors that the reason why graphene did not yet experience a takeoff in any technology is in a low quality of the commercially available graphene and in the lack of its derivatives with tailored properties^23^. The results of the systematic analysis of commercial graphene purchased from 60 different companies ended up with disappointing conclusions: material produced by the majority of companies contain no more than 10% of graphene; not a single company offers a material with more than 50% of pure graphene; not a single product contains more than 60% of sp^2^ carbon. To this end, a highly competitive plasma-based alternative that circumvents the drawbacks of conventional chemical technologies has been developed^24^.

It has been reported, for the first time, application of microwave-driven plasmas in atmospheric pressure for the single-step production of N-doped high quality free-standing graphene sheets^24–31^. The advantages of this approach are in its reproducibility and ability to control morphological, structural and functional properties of the synthesized material. The end-result is production of graphene sheets, highly doped by nitrogen (up to 8%at.), collected with ~ 40% in the form of single atomic layers, with very low-content of oxygen (< 1%) and high-ratio of sp^2^/sp^3^ carbons (~ 15)^24–31^. Coating of stainless steel and copper by this material has recently been performed using electrophoretic deposition, which reduced SEY of samples from about 2.4 to 1.0. Moreover, air exposure of such sample and/or high vacuum baking at about 150 °C for 64 h changes the SEY for just few percent^32^.

Secondary electron yield of graphene can be further improved by tailoring its electronic structure. Following the three-step model commonly considered describing the SEE, in the first step primary electrons impinging surface lose their energy inside the material mainly by excitation of valence electrons and the creation of plasmons^33^. Both processes contribute to the generation of internal secondary electrons by their direct excitation from the valence band or via Landau damping, respectively^34^. During the transport of internal secondary electrons through the material, which is the second step of SEE, energy loss processes again take place, so that only minority of electrons reaches the surface with sufficient energy to be emitted. The energy loss efficiency of primary and internal secondary electrons depends on the electronic structure, e.g. density of states in the valence band and above the vacuum level, or the ability for plasmon excitation. Doping of graphene by foreign atoms, such as nitrogen, modifies its electronic properties, and significantly affects graphene plasmon performance^22^. In addition to the usual for pristine graphene, π and π + σ plasmons, low-energy 2D-plasmons appear in doped graphene^22^. In this regard, the properties of N-graphene are conferred by N atoms within the carbon lattice, where they typically form pyridinic, pyrrolic, graphitic and oxidized nitrogen functional groups. For instance, it was reported that incorporation of nitrogen in the graphene scaffold affects the material work function, depending on the type of functional groups formed: graphitic nitrogen decreases work function, whilst presence of pyridinic and pyrrolic groups affects its increase^35^. N-doping shifts the Fermi level and opens a band gap near the Dirac point. Each graphitic N can contribute ~ 0.5 electron to π network of the graphene lattice, resulting in an n-doping effect. However, pyridinic and pyrrolic N are formed at the edges or defect sites. These defects impose the p-doping effect accompanied with a downshift of the Fermi level towards the Dirac point and withdrawing electrons from the graphene sheet, i.e., inducing electron-deficient nature of the valence band^36-38^.

Despite of the numerous approaches available for N-graphene synthesis, either by direct or post-synthesis treatment, many issues remain unsolved, such as the ability to control the type and level of doping. The direct CVD method, for instance, requires high temperature, vacuum systems and suffers from metal interference, low yields and high costs^39–42^. Other direct synthesis methods, such as ball milling, solvothermal synthesis, segregation growth, arc plasma, etc. have the potential to provide homogeneous doping, contrasting with post-treatment methods that provide surface doping only. Solvothermal approach typically involves mixing of lithium nitride with tetrachloromethane under moderate to high pressure and temperature that facilitate the interaction of precursors during the synthesis^43^. The method provides high yield and high doping levels but with very high oxygen content (larger than nitrogen one). Graphene oxide (GO) and nitrogen precursors (e.g. urea, NH3, etc.) are the initial materials for wet chemical methods and thermal treatment. The high temperature required for GO thermal treatment makes the process costly and inefficient. Although the wet chemical approaches can provide cost-efficient large-scale production, they rely on the use of harsh chemistry (e.g. strong acids)^44–48^. Moreover, these methods are based on lengthy/complex batch procedures that do not provide control over the assembly process. Plasma post-treatment methods offer short reaction times, low power consumption, but also low yield, inhomogeneous doping, and high level of oxygen content^20,41,47^. To foster N-graphene as a cost-effective material with low SEY several challenges need to be addressed. In the first place is development of a method for large-scale production of N-graphene relying on cost-effective and environmentally friendly synthesis processes, capable of providing controllable and reproducible nitrogen doping.

To this end, the synthesis of N-doped graphene through plasma treatment presents significant advantages relatively to other technologies because plasma systems simultaneously comprise thermal and chemical reactor functions along with catalytic properties. Moreover, it provides the ability to control the amount and localization of energy and matter delivered from plasma bulk to developing nanostructures at atomic scale level, the key to achieve the desired morphological, electronic and functional properties of targeted materials. In this work we intent to provide substantial evidence that microwave plasma technologies can be used as a competitive and disruptive alternative route to the chemical methods for the production of self-standing N-graphene sheets with controllable level and type of N doping, i.e. pirydinic, pyrrolic, graphitic, etc. A novel plasma approach is used to achieve in situ doping of the graphene sheets during their assembling and growth in the “mild” plasma zone of the reactor. The control on the type and level of doping has been achieved by changing of N-precursor injection position in the plasma environment, flux of the precursors, i.e. number density of N atoms as well as type of the N bonds configuration in the precursor utilized. Moreover, the possibility to tune the density and energy of the carbon building units, i.e. C_2_ radicals, and C atoms, in the high energy–density plasma environment, which translates in an effective control over the energy and material fluxes towards growing nanostructures in the assembly “mild” zone of the reactor, constitutes these methods’ most crucial advantage. Raman spectroscopy, scanning electron microscopy, high resolution transmission electron microscopy (HRTEM), X-ray photoelectron spectroscopy (XPS), X-ray diffraction (XRD) and Near Edge X-ray-absorption fine-structure spectroscopy (NEXAFS) were used to probe the morphological, chemical and microstructural features of the produced material. The secondary electron emission properties of synthesized free-standing N-graphene sheets with different level of doping and dominant pyridinic/pyrrolic N bonding configuration have been examined. The morphology of N-graphene sheets, characterized by wrinkles and lateral corrugations, contributes to suppression of secondary electron emission due to the efficient recapturing of emitted electrons. Furthermore, the changes in the electronic structure of graphene induced by well-controlled level and type of nitrogen doping, with the correspondent changes in the performance of graphene plasmons, have demonstrated the potential to translate in sub unitary (< 1) SEY values. Since the procedure for electrophoretic deposition of free-standing graphene that preserves its overall properties was already developed^32^, properly customized free-standing N-graphene with low SEY is a promising low secondary electron yield material of interest in accelerators and space technologies.

## Experimental

### The plasma process under consideration—N-graphene sheets in situ synthesis

**Figure 1:**
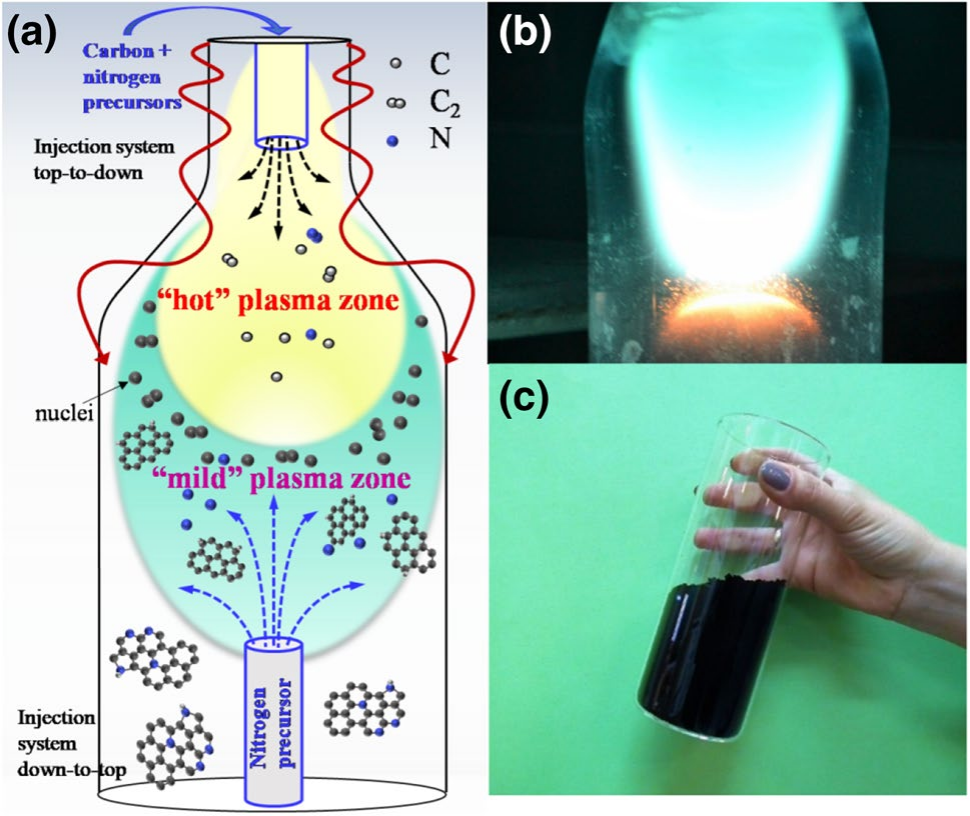
Schematic representation of plasma reactor (**a**); plasma delivering N-graphene sheets (flowing sheets are seen in yellow) (**b**); 1 g of N-graphene powder as synthesized (**c**).

The assembly of nanostructures is a chain of events that starts with the generation of precursor species and the formation of building units, their redistribution, clustering and formation of stable nuclei, followed by growth and structure formation. In order to achieve truly deterministic synthesis processes, an effective control over the most relevant self-organization pathways in plasmas is pursued. Prioritizing operational flexibility, a waveguide-surfatron based setup was used to create a surface wave (SW) induced microwave plasma at atmospheric pressure conditions^24^ (see supplementary material 1). The surface wave sustained discharge produced by the field of a travelling wave (represented by the red lines in the Fig. 1) has an extended active zone outside the wave launcher because the plasma is sustained by a wave that simultaneously propagates and creates its own propagation structure. In this way, large microwave power densities can be injected into processing area and high population densities of active species of interest can be achieved. The discharge takes place inside a quartz tube inserted perpendicularly to the waveguide wider wall and directed downstream as seen from Fig. 1a,b. The design permits control over the thermodynamic conditions (gas velocity, thermal fluxes, residence time etc.) in the plasma reactor.

The reactor can be virtually separated in three different zones as can be seen from the scheme presented in Fig. 1a. The first one is the surface wave sustained discharge zone, including the zone inside the launcher (not shown in the figure) and the extended "hot" plasma zone outside the launcher (Fig. 1a). Here, the wave power is absorbed primarily by plasma electrons, which transfer the power to heavy particles via elastic and inelastic collisions resulting in gas temperatures up to 3,000 K. The gas temperature gradually decreases moving away from the launcher and then drops in the "mild" plasma zone (20–30 cm)^24^. The Carbon precursor (e.g. ethanol) mixed with background argon gas is injected in the "hot" high energy density zone where decomposition processes of the injected carbonaceous molecules take place as a result of particles collisions and intensified chemistry. The created radicals become precursors of chain reactions leading to creation of the main building blocks, i.e. carbon atoms and C_2_ radicals. The next "mild" zone in axial (z) direction includes the "near" plasma afterglow where the gas temperature drops from about 3,000 to 500 K. It is important to note that the gas temperature changes also radially due to the radial thermal losses. The radial profile of the temperature is approximately parabolic. The transport of gas-phase carbon atoms/molecules into colder zones through the so-called vaporization surface^27^ results in transformation into solid carbon nuclei. An isothermal plasma surface of about 2,500 K is considered as a “boundary of vaporization”. The transport of plasma generated carbon atoms/molecules into colder zones (outside of the vaporization boundary) of the reactor results in formation of solid carbon nuclei that are gradually withdrawn in the outlet plasma stream where kinetic processes of assembly and growth of “flowing” carbon nanostructures take place. Given the fact that the nucleation and growth processes are determined by the interplay of kinetic and thermodynamic factors, the engineering of structural qualities of targeted nanostructures was achieved via synergistic tailoring of the "hot" plasma environment and thermodynamic conditions in the “mild" zone of the plasma reactor. Therefore, by tailoring the temperature gradients, the density of carbon molecules and atoms as well as their residence time, selective synthesis of graphene sheets in a narrow range of operational parameters can be achieved. The processes of assembly and growth of graphene sheets take place in the “mild” plasma zone, during their flight with background gas flow.

Two different approaches have been applied to inject nitrogen precursors. The so called top-to-down injection of nitrogen precursor consists in the simultaneous introduction of carbon and nitrogen precursors into the “hot” plasma zone as shown in Fig. 1a. In this case, using of methylamine as nitrogen precursor results in methylamine decomposition and formation of CN radicals (2CH_3_NH_2_ → 2·CN + 5H_2_) in the “hot” discharge zone. Furthermore, the formation of HCN as “building blocks” for N-graphene, is due to reactions such as, ·CN + H_2_ → HCN + H· and ·CN + H· → HCN, occurring in the “mild” plasma zone. Considering down-to-top injection, a gas jet containing the N precursor is sprayed in the “mild” zone (see Fig. 1a) against the main flux, and N containing radicals are burst out in the volume under consideration, resulting in a homogeneous distribution of the precursor units. Reaction involving CH_x_ and NH_y_ radicals results in the formation of highly reactive HCN molecules that bond to the flowing graphene sheets forming mainly “edge” pyridine and pyrrolic bond configurations^49,50^. Since the nanosheets are created in the plasma zone, where the electrons rapidly settle on the graphene surfaces, the sheets are negatively charged thus preventing their agglomeration. The configuration used allowed the control over the N bonding types as well as the level of doping via changing the position of the injection point in the “mild” plasma environment, number density of injected precursor units and by using gas/liquid precursors with different N types.

Using the reactor with an expanding radius allows large power densities inside the sections with smaller radius and to achieve larger fluxes of building units (C_2_, C) with sufficiently high energy towards "mild" plasma zone. Here, in the larger volume, the departure from the supersaturation conditions of the environment prone to foster nanosheets assembly is deliberately preserved^25,27,28^. Therefore, in the larger volume, the density of carbon nuclei decreases, and, as a result, the conditions favor the creation of planar nanostructures. Moreover, the assembling process requires very fast delivering and stacking of building units and equally fast supply of enough energy to overcome all the actual potential barriers. Thus, the fluxes were adjusted to satisfy conditions for well-tuned residence time of the flowing nanostructures in the “assembly” zone of plasma reactor.

Free-standing N-graphene sheets have successfully been fabricated at a relatively high yield (~ 0.5 mg/min) using ethanol and methane as carbon precursors and ammonia and methylamine as nitrogen precursors. It should be noted that the selective synthesis of N-graphene sheets can be obtained in a very narrow range of discharge operational conditions as previously reported^24^. In Fig. 1c, a container with 1 g of as-synthesized N-graphene is shown. The production cost of this black, light and fluffy material is estimated at ~ 160 € per gram, including electricity, cooling and used gases, which places the process very competitively in the N-graphene production market (about 5 times cheaper and with lower oxygen content than, for example, commercially available product from a reference supplier^51^).

## Results and discussion

### N-graphene–physico-chemical characterization

**Figure 2:**
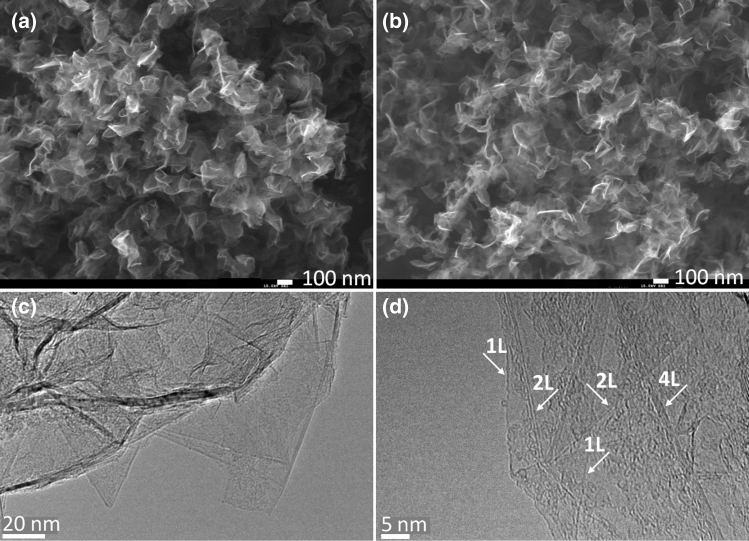
SEM images (× 40 000) of the as-synthesized N-graphene sheets (**a**) ammonia precursor (Q_Ar_ = 1,200, Q_Eth_ = 35 sccm, Q_Am_ = 50 sccm) in down-to-top scheme; (**b**) methylamine precursor (Q_Ar_ = 1,200 sccm, Q_Eth_ = 35 sccm, Q_Meth_ = 3 sccm) in down-to-top scheme; (**c**, **d**) HRTEM images of the N-graphene sheets with ammonia precursor; (number of (e.g.1L etc.) layers is indicated).

In the down-to-top scheme the nitrogen precursor injection point is in the “mild” plasma zone at a distance z = 12 cm from the launcher (Fig. 1a). SEM images (Fig. 2a,b) of N-graphene sheets fabricated under this approach using ammonia/methylamine as nitrogen precursors reveal the typical graphene morphology, i.e., disordered entangled sheets entangled. Sheets obtained with methylamine as nitrogen precursor are more congested.

Typical TEM images of N-graphene sheets at different magnifications are shown in the Fig. 2c,d, where thin sheets with sizes ranging from 100 to 200 nm can be seen. The sheets are folded, overlapped and wrinkled as evidenced by dark areas in the images. The clearer and homogeneous regions correspond to single-layer (or few-layer) sheets of N-graphene, as confirmed in the image with higher magnification (right). The visible curling at the edges of the sheets seen in the HRTEM images allows for an estimation of their thickness. These edges are revealed in TEM imagery as dark lines, and both single (1L) and multi-layers N-graphene structures can be identified in Fig. 2d. The highly ordered lattice fringes observed also indicate that the sheets are well-crystallized.

**Figure 3:**
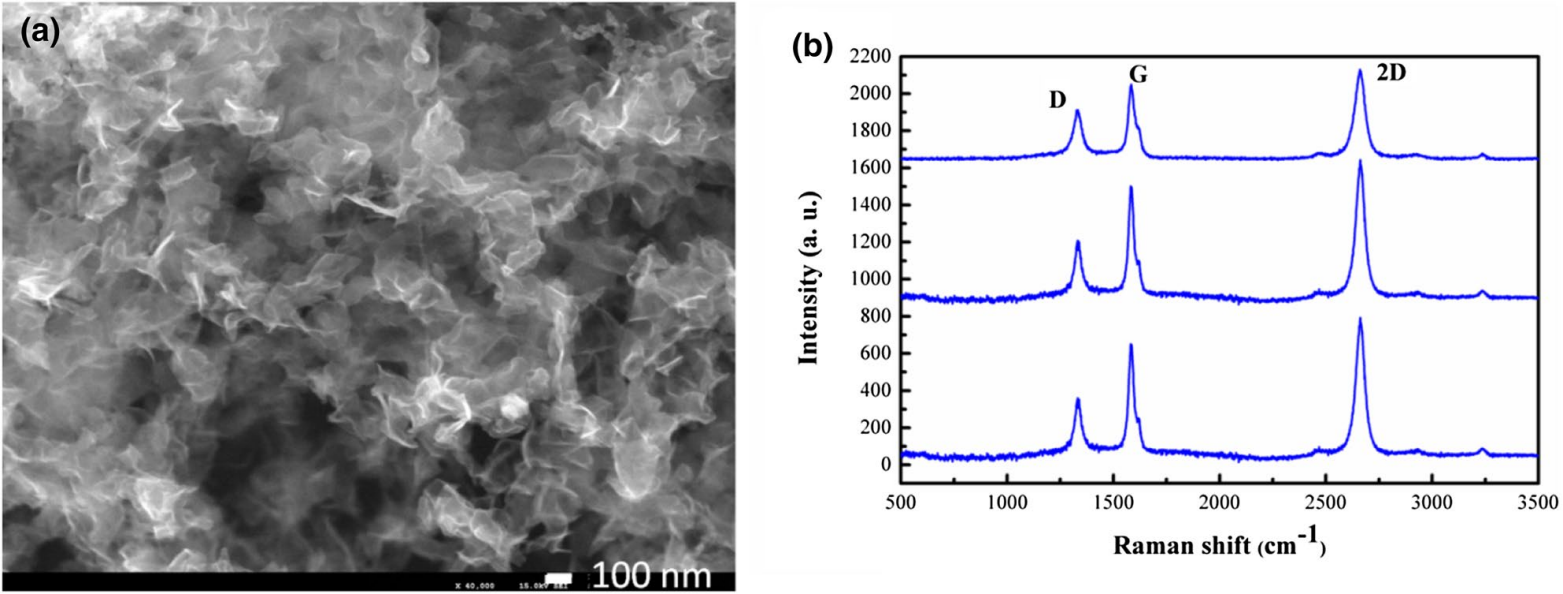
(**a**) SEM image (× 40,000) of N-graphene sheets and (**b**) corresponding Raman spectra obtained at three randomly chosen locations (Q_Ar_ = 1,200 sccm, P = 2 kW, Q_CH4_ = 20 sccm, Q_Meth_ = 6 sccm) in top-to-down scheme.

Furthermore, considering a top-to-down injection scheme, carbon/nitrogen precursors have been injected into “hot” plasma region. Typical graphene morphology and corresponding Raman spectra measured at three randomly chosen sample regions are shown in Fig. 3a,b. All Raman spectra are characterized by the presence of the peaks attributed to the D, G and 2D bands, located at 1,333 cm^−1^, 1,584 cm^−1^, and 2,662 cm^−1^, respectively. The G-band results from in-plane vibrations, present in all sp^2^ carbon structures, while other lines are manifestations of one-phonon forbidden scattering and/or two-phonon resonant scattering. The D-band arises from a breathing mode of sp^2^ carbon rings and it is activated by defects. The defects can originate from hybridization, bond-angle and bond-length disorders, dangling bonds at the flake edges and incorporation of nitrogen atoms. The 2D-band is the most prominent feature in the Raman spectrum of graphene, being regarded as a fingerprint for graphene. It is the result of two-phonon scattering. The intensity of 2D peak in the recorded spectra evidences the high quality of the N-graphene structures. The ratio of D to G peak intensities remains nearly constant (~ 0.6) across different locations, thus indicating nearly homogeneous nitrogen doping of graphene structure. The 2D to G peak intensities ratio (~ 1.2) and the full width at half maximum of the 2D-band (~ 52 cm^−1^) illustrate that the sample contains graphene sheets composed by few monolayers^52^. Synthesized free-standing graphene sheets contain few atomic layers, as estimated from the Raman and TEM results. Having in mind that the interlayer distance in the sheets is about 3.46 Å (as shown by XRD) the thickness of these structures is about 1–2 nm.

**Figure 4:**
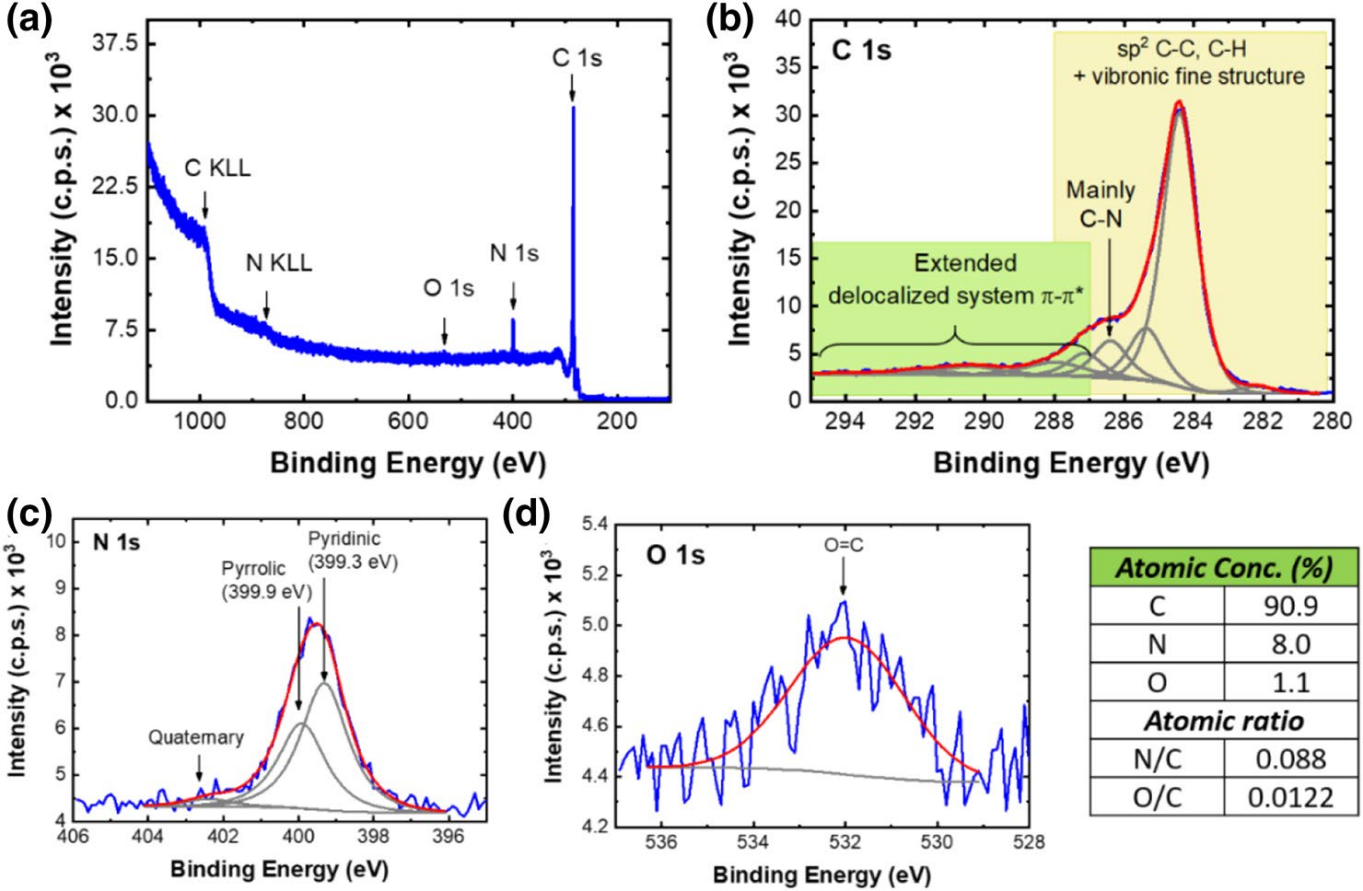
(**a**) Survey XPS spectrum; (**b**) C1s region; (**c**) N1s region, (**d**) O1s region of sample synthesized with methylamine (Q_Ar_ = 1,200 sccm, Q_CH4_ = 20 sccm, Q_Meth_ = 6 sccm) in top-to-down scheme.

**Figure 5:**
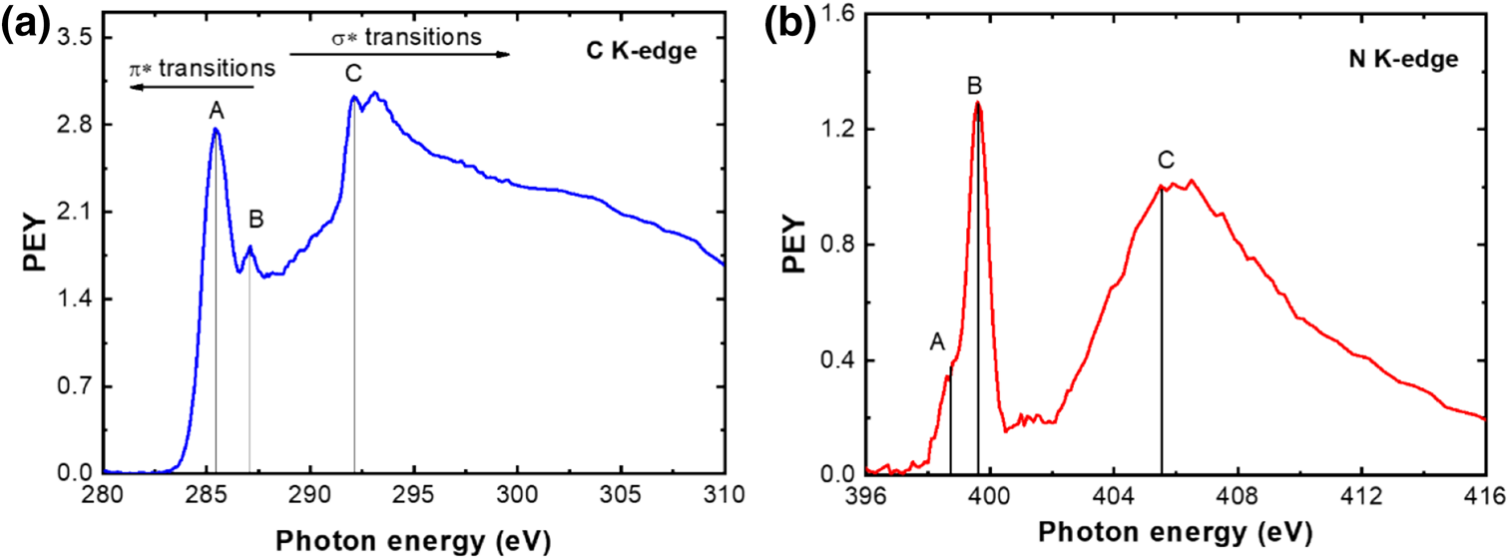
(**a**) C K-edge NEXAFS spectrum; (**b**) N K-edge spectrum of sample synthesized with methane/methylamine precursors (Q_Ar_ = 1,200 sccm, Q_CH4_ = 20 sccm, Q_Meth_ = 6 sccm) in top-to-down scheme.

The use of methane/methylamine as carbon/nitrogen precursors significantly increases the graphene doping level. The survey XPS spectrum (Fig. 4a) shows the C 1s, O 1s, and N 1s photoelectron peaks, as well as the carbon and nitrogen Auger structures C KLL and N KLL. As it can be seen, the N 1s peak intensity is much larger than that of O 1s. As for the most prominent feature, the C 1s region, its main contribution comes from the sp^2^ carbons, while the peak fitted at ~ 286 eV is mainly attributed to carbon bound to nitrogen^53^ (Fig. 5b). The N 1s region (Fig. 5c) was fitted with two main peaks, centered at 399.3 eV and 399.9 eV, assigned to *pyridinic and pyrrolic nitrogen atoms*, respectively^53^. Centered at 402.4 eV *quaternary nitrogen atoms* are detected^54–56^. Figure 4d shows the oxygen region in more detail from which *a residual Oxygen Atomic Concentration (1.1%)*, is quantified. Therefore, N-graphene prepared with methane/methylamine, holds a *high nitrogen doping level (Atomic Concentration* = *8%).* The obtained results reinforce the advantages of the novel plasma-based method as compared with the chemical ones. The graphene lattice structure containing more than 60% sp^2^ carbons is well preserved after doping and the oxygen level is kept at residual amount, by contrast with the materials synthesized via chemical methods.

Furthermore, the Near Edge X-ray-absorption fine-structure (NEXAFS) spectra, presented in Fig. 5a,b, were obtained on the C K-edge and N K-edge. As seen, characteristics sharp C 1s → π* resonance at ~ 285.5 eV^57,58^ and the σ* resonance at ~ 292 eV^57,59^ are detected. Additionally, a peak at ~ 287 eV is observed, which is attributed to C=N bond^60^. A pronounced peak is detected in N K-edge spectrum (Fig. 5b) indicating a relatively high nitrogen doping level, thus compatible with the XPS results. A narrow peak at 399.6 eV (B) is usually assigned to pyrrole/amino groups/substitutional graphite-like bonding^60-62^. Other contributions at 398.8 eV (A) and 402–410 eV (C) are attributed to pyridine-like bonding that arises from transitions from the K-shell to the unoccupied π* orbital^62-64^ and to N–C/N–H bonding^62–65^, respectively.

X-ray diffraction analysis made shows that interlayer spacing is about 3.46 Å and exhibits small variations with level of doping (see supplementary material). The measured levels of interlayer spacing are larger than those of graphite (3.35 Å) is to be noted.

### Electrical conductivity of the synthesized N-graphene sheets

**Figure 6:**
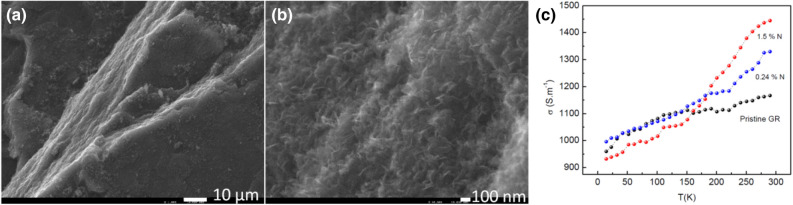
SEM image of the cross section (**a**) of a tablet showing in-plane ordering of N-graphene flakes in platelets and (**b**) image with higher magnification where disordered N-graphene sheets on the top are clearly seen; (**c**) Dependence of N-graphene conductivity on the temperature.

In order to assess the electrical conductivity of the graphene sheets and its temperature dependence, 0.1 g of the N-graphene powder was pressed into disc pellets of 8 mm diameter and about 3 mm of thickness. The electrical conductivity was measured applying the Van der Pauw method^66^. The measurement geometry utilizes a four-contact scheme, where pinching point contacts are located on the periphery of the pellet. This design provides measurement of the longitudinal conductivity as far as the current lines are parallel to the pellet surface. Due to the compressing, the individual sheets have parallel orientation, as seen in Fig. 6.

Figure 6c presents typical temperature dependence of the electrical conductivity of samples with 0.24 and 1.5 at N % in the temperature interval from 300 down to 10 K. Pristine graphene is also shown for comparison. High values of the electrical conductivity in the order of 10^3^ S m^−1^ are observed at temperature of 300 K. However, the real electrical conductivity should be even higher, considering that the measurements were performed on compressed disks made from individual N-graphene sheets, where a conductive network is formed through the matrix of highly conductive sheets having multiple interfaces between them.

The temperature characteristics show thermally activated transport and non-metallic behavior. As resistance increases, conductivity decreases accordingly at decreasing temperature (i.e. dσ/dT > 0), as opposed to typical metal behavior. The measured σ(T) values increase by 65% as temperature T increases from 10 to 300 K. Typical insulators (high work function) or localized systems have dσ/dT > 0. Graphene is a rather unusual electronic material that often exhibits dσ/dT > 0 at certain carrier densities and temperature range while showing the expected "metallic" behavior dσ/dT < 0 in other density and temperature ranges^67^. A non-monotonic temperature dependence in the measured electrical conductivity and “insulating” behavior at low temperatures is reported for CVD grown monolayer graphene^68^ and bilayer graphene^69^. The anomalous transport dependence on temperature in graphene is theoretically studied as arising entirely from disorder effects^70^. It should be noted that the graphene samples studied consist of compacted graphene sheets and therefore, the temperature dependencies σ(T) could be analyzed in the frame of disordered systems not being the aim of this investigation. We should note that the room temperature values σ (300 T) reveal explicit influence of the N doping content, with the higher atomic percentage showing higher conductivity.

### Secondary electron emission of N-graphene sheets

Considering the morphology and the high electrical conductivity, the properties of synthesized N-graphene flakes as a low secondary electron emission material have been investigated. Moreover, the typical graphene crumpled nature, yielding in highly corrugated surface morphology, can also contribute to reduce SEY. The SEY values of Highly Oriented Pyrolytic Graphite (HOPG) were used as a reference, since secondary electron emission properties of HOPG are well known.

SEY measurements in the 40–1,000 eV have been performed on a home-made experimental setup similar to the one described in^71^. The setup consists of an electron gun and a large Faraday cup, connected to an electrometer. The sample is mounted on a sample holder placed inside the Faraday cup, in front of the entrance aperture. All measurements have been performed at normal incidence. The sample holder is galvanically decoupled from the Faraday cup. On the entrance of the Faraday cup there is a suppression electrode in the form of an aperture, kept on the potential of − 20 V, in order to reflect the electrons that would otherwise escape. When the sample is in shortcut with the Faraday cup, primary electron beam current I_p_ is measured. When the sample is biased negatively to − 15 V, the current of all electrons leaving the sample surface I_tot_ is measured. Therefore, the ratio between the two currents is the total electron yield although its major part is secondary electron yield, and it is calculated by the following ratio:

$$ {\text{SEY}} = {\text{I}}_{{{\text{tot}}}} /{\text{I}}_{{\text{p}}} $$

It should be noted that SEY measured in this way encompasses not only so-called true secondary electrons (with kinetic energies bellow 50 eV), but also those that are elastically and inelastically backscattered.

To restrict the study only to the graphene/N-graphene properties as low SEE materials, the graphene samples for SEY measurements were prepared by powering small quantity of graphene/N-graphene powder on an indium plate, which is then placed in-between two pieces of aluminium foil. Applying pressure of ~ 105 Pa on the plate provides enough adhesion between graphene and indium, while the surface morphology of the graphene sample is not affected. The estimated thickness of such graphene patch is few hundred micrometres, so that primary electrons cannot reach the substrate.

**Figure 7:**
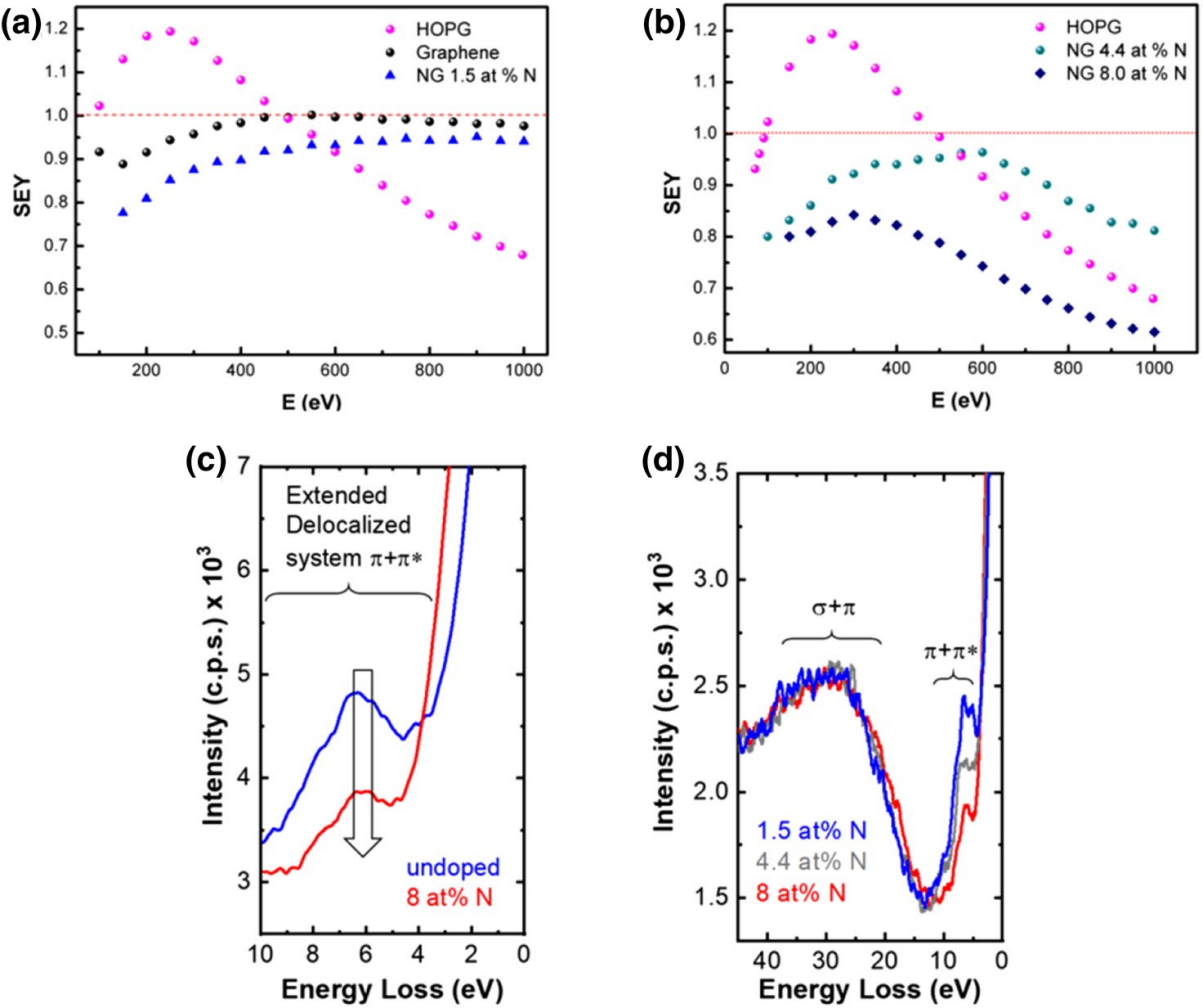
SEY vs energy plots for pristine graphene, N-graphene and of HOPG (**a**); for N-graphene with different levels of doping 8 (**b**); Energy loss features of C 1s XPS spectra of pristine Graphene and N-Graphene (8 at % N) (**c**); Energy loss features of C 1s XPS spectra of N-graphene at different levels of doping (**d**). The plots were obtained after normalizing to the C 1s baseline intensity and shifting the spectra of − 284.4 eV (which corresponds to sp^2^ C–C and C–H).

The SEY curves for HOPG graphene and N-graphene samples with different levels of N-doping are shown in Fig. 7a,b. HOPG is known as a low SEY material with typical maximum values falling in the 1.2–1.3 range that is in agreement with our measurements. Its SEY curve exhibits a well-defined maximum at about 300 eV followed by fast drop, which is characteristic of perfectly flat surfaces. SEY values for Graphene/N-graphene samples are bellow one, with N-graphene performing better than the pristine graphene (synthesized via the same procedure used for N-graphene, using only the carbon precursor). As shown, the shape of SEY curves for pristine graphene and N-graphene with low level of doping, i.e. 1.5 at.% N, differ significantly from the HOPG one. SEY initially increases with primary electrons energy and remain practically flat after reaching the maximum value. This shape is characteristic of a highly corrugated surface morphology^32^. The pristine graphene morphology is similar to that of N-graphene with low level of doping as evidenced by SEM images (see Figs. 3, 4) in^24^. This type of morphology contributes to a reduction of the SEY for graphene/N-graphene relatively to HOPG. However, the maximum SEY for pure graphene sheets is 1 while for N-graphene with level of doping 1.5 at N% is about 0.95. These results indicate that the lower SEY of N-graphene can be related to the change in the electronic structure of graphene caused by N-doping. The latter has a complex influence on secondary electron emission process.

Furthermore, increasing the N-doping level results in a significant change of the SEY curve shape. The shape of N-graphene SEY curves resemble the HOPG one but the maximum SEY is reduced to 0.84 for N-graphene with 8 at.% N, as seen from Fig. 7b. For 4.4 at % N, the SEY peaks at 0.96, keeps nearly flat in the energy range between 300–600 eV and then drops, following the same behavior of the HOPG curve. The obtained results demonstrate lower (below 1) SEY values for the N-doped versions of graphene, with further decreasing as the doping level increases up to 8 at% N. All the measurements have been carried out with as synthesized materials, i.e., no additional processing has been applied to the samples.

What is the reason for the SEY decrease as the N doping increases? The secondary electron emission is typically described as a 3-step process. In the first step, primary electrons impinging the surface lose their energy by exciting the electrons in the bulk. The energy of primary electrons is spent on the formation of “internal” secondary electrons. There are three mechanisms of creation of “internal” secondary electrons: (a) excitation of inner shell electrons in electron-atom collisions (which does not depend on electronic structure of the material considered), (b) excitation of valence electrons and (c) via excitation of bulk and surface plasmons, i.e., collective oscillations of valence electrons excited by primary electrons during their penetration into the material. The plasmon decay results in the excitation of single valence electrons, i.e. creation of electron–hole pairs in the process of Landau damping^34^. The contribution of the last two processes is comparable and strongly depends on the valence electron density^22,33,34,72,73^. The valence electrons excited by direct electron–electron momentum transfer can acquire a variety of energies above the vacuum level E_vac_, while the electrons picking up the energy of decaying plasmons will always get the same amount of energy. Most of the secondary electrons originate from the valence band. In the second step, these “internal” secondary electrons lose energy during their transport towards the surface and only the electrons created nearby the surface with enough energy to overcome the surface energy barrier can be emitted. The third step is emission, where trajectory refraction and quantum reflection on the barrier are in play. Hence, in the case of conductive materials, high SEY is secured with high concentration of valence electrons excited in the near-surface region.

SEY values measured for N-doped graphene are consistently lower than the ones obtained for pristine graphene. Therefore, doping seems to open another energy loss mechanism that does not contribute to the internal secondary electron production, leading to a “waste” of a fraction of primary electrons. Therefore, the modification of the electron structure due to the incorporation of foreign N atoms, that have a quite complex influence on SEE, should be strongly related with reducing the sources of internal secondary electrons. The two internal secondary electrons creation channels, i.e. direct electron–electron momentum transfer and plasmons decay, depend on the density of valence electrons. As shown in a previous section, the predominant nitrogen functional groups are pyridinic and pyrrolic. Pyridinic/pyrrolic bonding configurations impose p-doping effect accompanied with a downshift of the Fermi level towards the Dirac point. As a result, withdrawing of electrons from the graphene sheet appears, thus inducing electron-deficient nature of the valence band. Moreover, plasmons decay is strongly influenced by nitrogen doping. Pristine graphene has the so-called π- and σ + π-plasmons with energies in the range (4.5–7 eV) and (14.5–27 eV), respectively. Both plasmon types have sufficient energy to generate internal secondary electrons in the process of Landau damping. A detailed comparison of the energy loss features in C 1s XPS spectra of pristine and N-graphene is shown in Fig. 7c. The energy losses assigned to π–π* excitations, i.e. π-plasmons are detected roughly between 3 and 10 eV (Fig. 7c)^29,53^. As seen, this peak is strongly decreased for N-graphene, which in turn results in lower production of internal secondary electrons via plasmons decay. In fact, the π–π* intensity decreases for higher doping levels as seen in Fig. 7d. The relative intensities of σ + π plasmon excitations, in particular the bulk plasmon losses, detected between 25 and 30 eV^29^, remain nearly the same regardless of the relative amount of N. Furthermore, a very small shift of π electron delocalization towards lower energies is observed for N doped graphene (see Fig. 7c). Such reduction of π-plasmon energy results in a decrease of the kinetic energy of internal secondary electrons generated by plasmons decay, which reduces their probability to reach the surface with kinetic energy above E_vac_. In addition, reducing kinetic energy of secondary electrons when they approach the surface lowers their transmission probability on the surface potential barrier. Both effects contribute to the decrease of SEY. Moreover, the presence of dopants may result in excitation of so-called 2D plasmons, with the energy of about 2 eV^22^. Due to the lower energy these plasmons cannot contribute to the production of internal secondary electrons and will further reduce the SEY. Therefore, the observed energy loss feature of C 1s XPS spectra give some confidence of the hypothesis that nitrogen doping of graphene reduces the contribution of source channel related with secondary electron production via π-plasmons decay.

**Figure 8:**
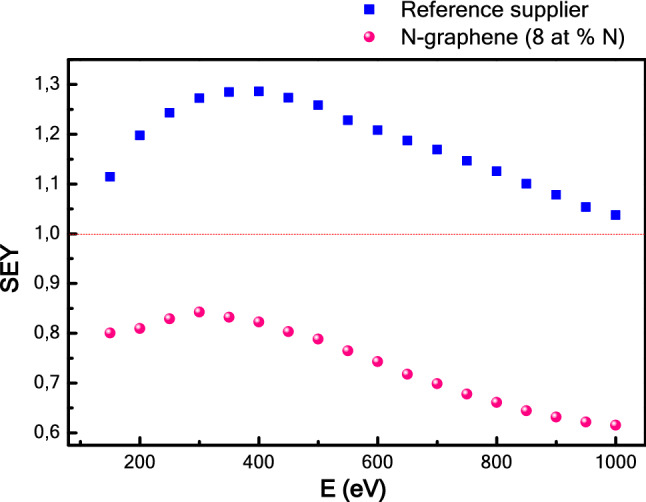
SEY vs energy plot for carbon material obtained from a reference supplier and for N-graphene produced in the context of this work.

Furthermore, to demonstrate the potential of plasma-produced N-graphene as prospective low SEE material, a comparison with commercial graphite powder has been carried out. The results demonstrate that the SEY values are about 1.5 times lower for N-graphene SEY when compared with those of commercial graphite powder from a reference supplier (Fig. 8). Maximum SEY values of the synthesized materials, similar commercial materials measured in this work and literature data are summarized in a Table 1.

**Table 1:** Maximum SEY values of different materials.

Sample/material	SEY_max_
HOPG	1.2
Amorphous carbon, CERN^9^	0.93–1.03
Graphite powder, reference supplier	1.3
Graphene deposited on flat Al (no detailed info)^21^	0.6–0.8
Graphene (this work)	1.0
NG 1.5% N	0.95
NG 4.4% N	0.96
NG 8.0% N	0.84

## Conclusions

This work shows that N-graphene, in particular N-graphene with predominant pyridinic/pyrrolic bonding configurations, can be exploited to reduce the SEE of carbon materials. A novel plasma based method was applied to achieve in situ at a single step doping of the graphene sheets during their assembly and growth in the “mild” plasma zone of the reactor. The control on the type and level of doping has been achieved by adjusting the position where the N-precursor is injected in the plasma environment and the flow of the nitrogen precursors.

The unique plasma ability to control the amount and localization of energy and matter delivered from the plasma bulk to the developing nanostructures is the key that has been applied to implement synthesis-by-design, at atomic scale level. The obtained results demonstrate that microwave plasma-based technologies are a valid alternative approach for the production of high-quality N-graphene with controlled morphologies and structural qualities and at high yields. Our solution is simple to use, versatile and scalable, meeting the requirements of the most exigent graphene-enabled product developers. The main advantage of the used synthesis method is the achievement of a very high and extremely controllable energy density in the plasma reactor, which allows effective control over the energy and material fluxes towards growing nanostructures at the atomic scale via proper reactor design and tailoring of the plasma environment in a synergistic way. The end-result is a high-quality product, obtained in a reproducible manner with the desired morphological, structural and functional properties. The estimated cost for 1 g of N-graphene with controllable doping level ( up to 8 at % N and oxygen in residual amount ~ 1%) and required doping configurations is about 160 €, much less than the prices of high-quality N-graphene available on the market (about 1,200 € per gram), while exhibiting much lower SEY values than those of commercial carbons from reference suppliers. The synthesis method shows potential for controllable, large-scale fabrication of other graphene derivatives, while promoting microwave plasmas as a competitive, green, and cost-effective alternative to presently used chemical methods.

Furthermore, the possibility to tune or suppress SEE is of fundamental importance in fostering N-graphene’s potential as a key player in improving the performance of low SEE coatings while preserving the properties of the substrates. The synergistic effect of the electronic structure modification and changes in the graphene plasmons behavior that result from the introduction of N atoms in the graphene scaffold, along with the peculiar “crumpled” morphology of the material lead to a decrease in the SEE.

These results indicate that plasma-synthesized N-graphene can be considered as a prospective low secondary electron emission material. The demonstrated possibility to tune graphene plasmons behavior is particularly important in improving the performance of plasmonic materials and in inspiring innovative solutions.

## Supplementary information

Supplementary information.
