# Supplementary material for: Prospects for microwave plasma synthesized N-graphene in secondary electron emission mitigation applications
Source: Sci Rep. 2020 Aug 3;10:13013. doi: 10.1038/s41598-020-69844-9 (PMC7398926; doi:10.1038/s41598-020-69844-9)
Supplement: Supplementary file 1 — Supplementary information. [file 41598_2020_69844_MOESM1_ESM.docx]

**Prospects for microwave plasma synthesized N-graphene in Secondary Electron Emission mitigation applications**

N. Bundaleska^1^, A. Dias^1^, N. Bundaleski^2^, E. Felizardo^1^, J. Henriques^1^, D. Tsyganov^1^, M. Abrashev^3^, E. Valcheva^3^, J. Kissovski^3^, A.M. Ferraria^4^, A.M. Botelho do Rego^4^, A. Almeida^5^, J. Zavašnik ^6^, U. Cvelbar^6^, O.M.N.D. Teodoro^2^, Th. Strunskus^7^ and E. Tatarova^1^

*^1^Instituto de Plasmas e Fusão Nuclear, Instituto Superior Técnico, Universidade de Lisboa, Lisboa-1049, Portugal*

*^2^CEFITEC, Departamento de Física, Faculdade de Ciências e Tecnologia, Universidade Nova de Lisboa, 2829-516 Portugal*

*^3^Faculty of Physics, Sofia University, 1164 Sofia, Bulgaria*

*^4^BSIRG, iBB, DEQ, Instituto Superior Técnico, Universidade de Lisboa, Lisboa 1049-001, Portugal*

*^5^Centre of Physics and Engineering of Advanced Materiais, Instituto Superior Técnico, Universidade de Lisboa, 1049-001, Lisboa, Portugal*

*^6^Department of Gaseous Electronics F6, Jozef Stefan Institute, Ljubljana 1000, Slovenia*

*^7^Institute for Materials Science, Christian Albrechts Universitaet zu Kiel, Kiel, Germany*

**Supplementary Material**

**Experimental setup and synthesis method**

A surfatron-based setup was used to create a surface wave induced microwave plasma at atmospheric pressure conditions as shown in Fig. 1^1^. The microwave power is provided by a 2.45 GHz generator (Sairem), whose output power was set to 2000 W. The generator is connected to a waveguide (WR-340) system, which includes an isolator, directional couplers, a 3-stub tuner and a waveguide-surfatron as the field applicator. The system is terminated by a movable short-circuit. The discharge takes place inside a quartz tube reactor, which is inserted downstream vertically and perpendicularly to the waveguide wider wall. The quartz reactor comprises two sections; a small one with internal and external radii of 0.75 cm and 0.9 cm, respectively, connected via conical section to the large tube with internal and external radii of 2.15 cm and 2.3 cm. The background argon gas is injected, under laminar gas flow conditions, into the discharge tube. Part of the background Ar gas flow passes through a tank filled with the carbon precursor, placed inside of sonication bath, with accurate control of the temperature to drag the ethanol molecules. A second quartz tube (internal and external radii of 0.5 cm and 0.35 cm, respectively), inserted inside the reactor tube, delivers the carbon precursor in the “hot” plasma zone. Two different strategies for nitrogen precursor entrance are used, i.e., spraying of the nitrogen precursor *top-to-down* into the “hot” and *down-to-top* into the “mild” zones of the plasma environment, respectively. Gas flow rates are controlled by a MKS247 Readout coupled to two MKS flow meters.

Selective synthesis of graphene sheets can be obtained in a narrow range of operational parameters^2^. For this reason, the experiments are all carried out under similar conditions, i.e. microwave power delivered to the plasma (P = 2 kW), argon flow (Q_Ar_ = 1200 sccm) and carbon precursor (ethanol) flow (Q_Et_ = 35 sccm), the flow of nitrogen precursors being the only variable parameter. The results considered are obtained with ammonia (Q_Am_ = 50 sccm) and methylamine (Q_M_ = 6 sccm) flow rates in *down-to-top* injection scheme. Considering *top-to-down* approach, the nitrogen precursor (methylamine) is sprayed from bellow using the small quartz tube inserted in the reactor. The injection point is located in the “mild” plasma zone at a distance z = 12 cm from the launcher (Fig 1). In this case methane (Q_CH4_ = 20 sccm) and methylamine (Q_M_ = 6 sccm) are used. The nanostructures are captured by a tornado-type system followed by a water trap to hold the nanostructures that are able to escape with the gas flow.


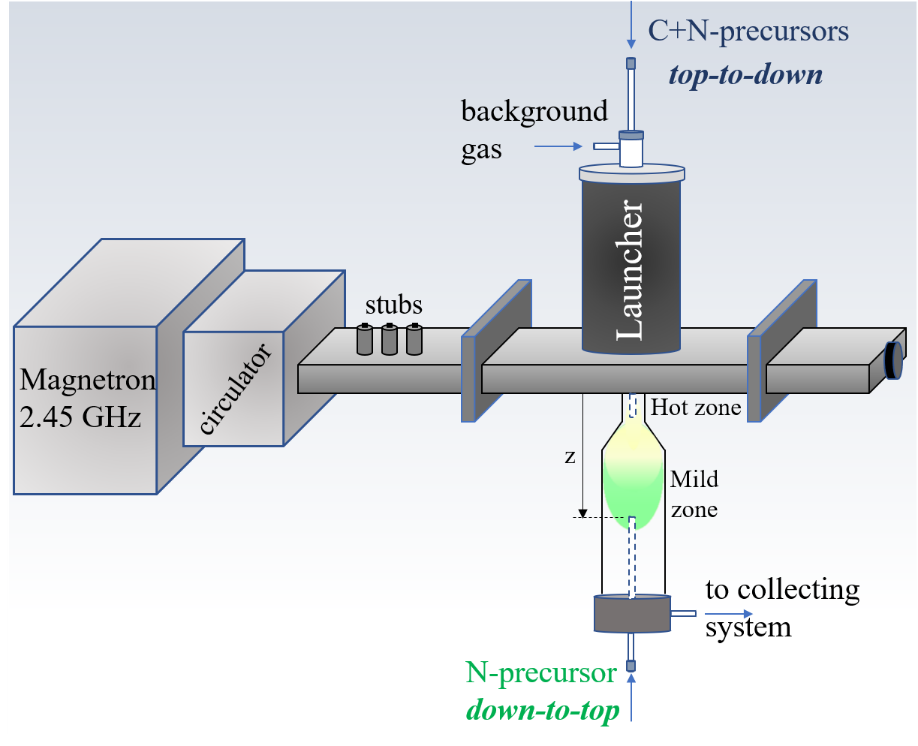


***Figure 1.*** *Experimental setup.*

**Characterization details**

**SEM** characterization of the samples has been performed using a JEOL, JSM-7001F field emission gun scanning electron microscope operating in secondary electron imaging mode (SEI) using 15kV accelerating voltage. The samples were deposited on a double-sided carbon tape mounted on an aluminium stub.

**HRTEM** The graphene flakes were placed directly as a solid powder onto a copper grid for transmission electron microscopy (TEM). The sheets were then characterized by a high-resolution TEM JEOL JEM-2010F, operating at 200 kV accelerating voltage.

**Raman spectroscopy** In order to perform the Raman spectroscopy characterization, the synthesized powder nanostructures were freely suspended on a glass plate and the Raman spectra from different regions on the sample were obtained with a LabRAM HR Visible (Horiba Jobin-Yvon) Raman spectrometer using 633 nm, 514 and 458 nm laser lines with 5 cm^-1^ spectral resolution and a laser spot size of 2 μm. Measurements were performed with a laser power *P* = 0.054 mW to avoid overheating.

**X-ray photoelectron spectroscopy** N-doped graphene sheets were characterized as-received by X-ray photoelectron spectroscopy using a XSAM800 spectrometer from KRATOS, operating in Fixed Analyzer Transmission (FAT) mode and a pass energy of 20 eV. Non-monochromatic X-rays were produced with a Mg Kα source (1253.6 eV), with a power of 120 W. Fluffy powder samples were mounted on a XPS holder with a double face tape. No silicon was detected discarding any interference from the tape spectrum. Samples were analyzed at room temperature, at UHV pressure around 10^−7^ Pa and a take-off angle set to 45º. The spectra were collected with 0.1 eV steps, using the software Vision 2 for Windows, Version 2.2.9 (from KRATOS). X-ray source satellites were subtracted. Shirley backgrounds and Gaussian/Lorentzian (GL) line shapes were fitted using XPS Peak 4.1 (freeware). N 1s was fitted with pseudo-Voigt profiles with Full Width at Half Maximum (FWHM) = 1.5 eV and GL = 85 % (L %); C 1s was fitted with pseudo-Voigt profiles with FWHM = 1.2 eV, for photoelectron peak components (roughly between 283 eV and 288 eV), and with FWHM = 2.0 eV, for energy losses peaks (which spread through an indefinite energy range depending on the extension of the electron delocalization, in this case roughly from 287.5 eV to 294 eV). In C 1s, GL = 65 % for all fitted peaks. No flood gun was used for charge compensation. The charge shift was corrected using the binding energy (BE) of aromatic C-C or C-H in graphene, centered at 284.4 eV. The sensitivity factors (from Vision 2 library) used for quantification purposes were 0.318 for C 1s, 0.736 for O 1s and 0.505 for N 1s.

**NEXAFS** Near Edge X-ray-absorption fine-structure (NEXAFS) spectroscopy was performed on the samples using the HE-SGM beam line at BESSY II storage ring (PREVAC end station provided by Professor Ch. Wöll). NEXAFS measurements were performed considering different incident angles (20-90°) relative to the substrate surface. The data was collected in respect to the C K-edge to characterize the carbon chemistry of the produced free-standing graphene, in the partial electron yield (PEY) mode (few nm depth), using a home built double channel plate detector. The energy resolution was ≈0.40 eV. The raw NEXAFS spectra were corrected for the beam line transmission by division through a spectrum of a clean, freshly sputtered Au sample. Alignment of the energy scale was accomplished by using an I0 feature referenced to a C 1s → π* resonance at 284.9 eV from a fresh surface of a graphite foil standard sample.

The temperature dependence of electrical conductivity of graphene sheets was measured in the temperature range 10 K < T < 300 K. Measurements were done in the dark using HP4140B pA-meter/dc voltage source. The samples were placed in a closed cycle ARS helium cryostat working in the temperature interval from 300 down to 10 K. To measure the electrical conductivity of the graphene sheets, N-graphene powder was pressed into discs pellets of 8 mm diameter and 1.2 mm thickness. The electrical conductivity was measured applying the Van der Pauw method^3^.

**Results – material characterization**


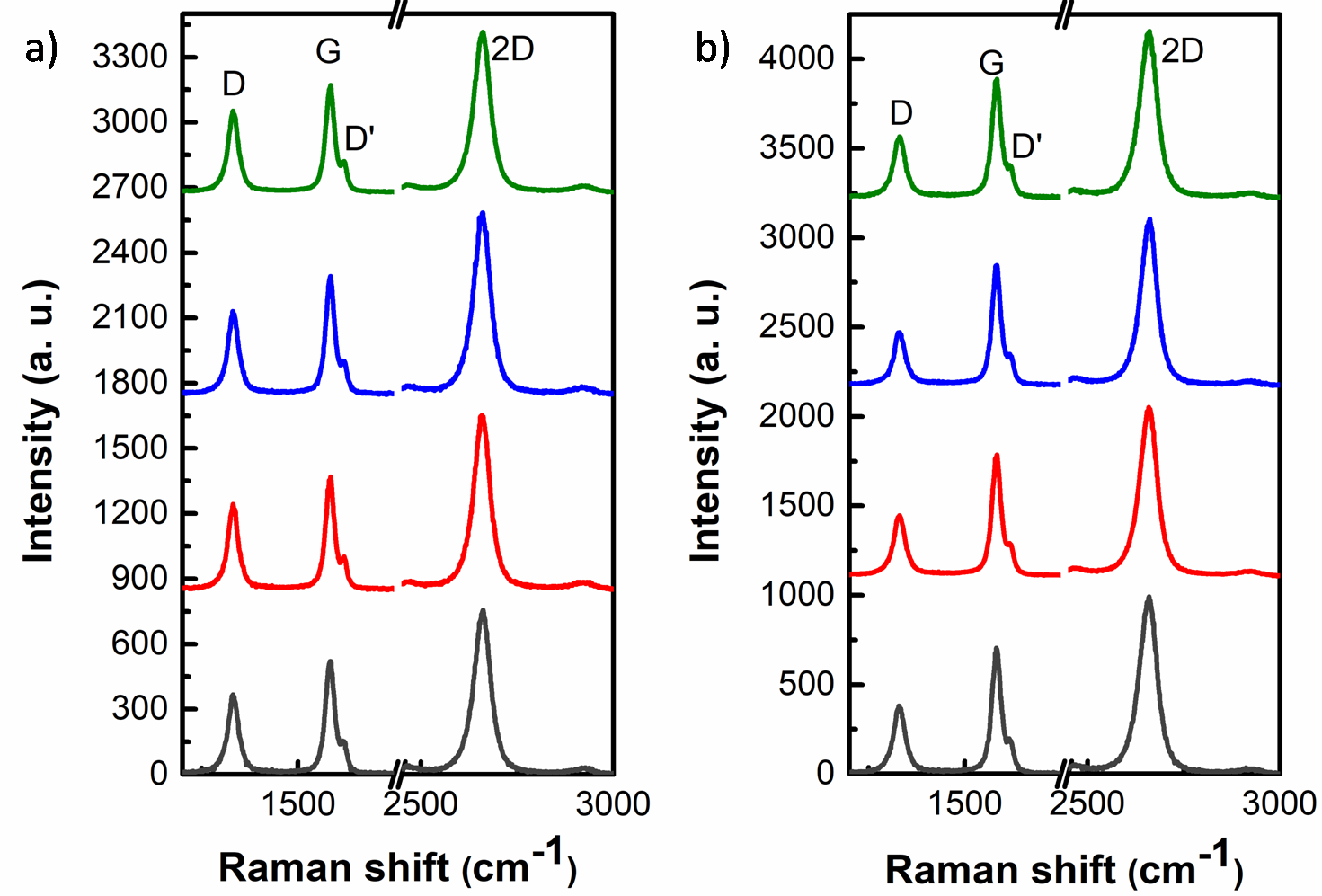


***Figure 2****.* *Raman spectra of N-graphene sheets produced with ammonia precursor (Q_Ar_ = 1200, Q_Eth_ = 35 sccm, Q_Am_ = 50 sccm) in down-to-top scheme; (b) methylamine precursor (Q_Ar_ = 1200 sccm, Q_Eth_ = 35 sccm, Q_Meth_ = 3 sccm) in down-to-top scheme.*

Raman analysis provides information about the N-graphene structural characteristics^4,5^. The Raman spectra of synthesized N-graphene sheets are shown in Figure 2. The spectra were collected at different spots in the samples and they consist of three dominant peaks at ~ 1332 cm^−1^, 1583 cm^−1^, and 2658 cm^−1^ attributed to the D, G and 2D bands, respectively. Given the fact that the ratio of D to G peak intensities keeps nearly constant (~0.7 and ~0.5 for ammonia and methylamine respectively) from different locations, homogeneous nitrogen doping may be considered. Taking into account the ratio between the 2D and G peak intensities (~1.4) and the full width at half maximum of the 2D-band (~48 cm^-1^) in both cases, the obtained results show that the samples contain graphene sheets with single or few mono-layers, that is also confirmed by HRTEM results.

*
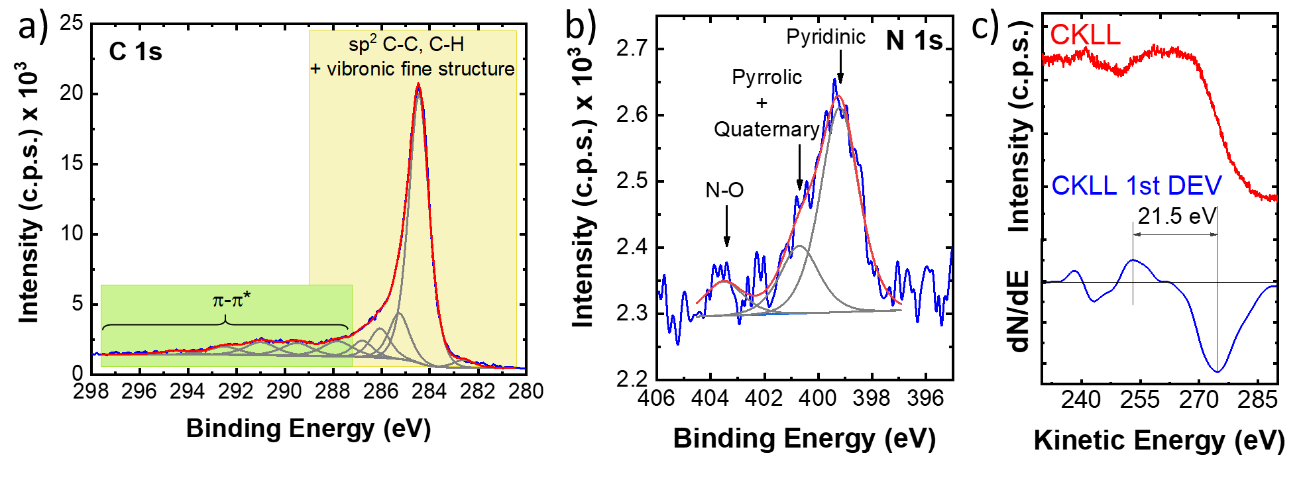
*

**Figure 3.** XPS regions: C 1s (a) and N 1s (b); Auger C KLL structure and respective 1^st^ derivative (c) (Q_Ar_ = 1200, Q_Eth_ = 35 sccm, Q_Am_ = 50 sccm) in down-to-top scheme.

To study the elemental composition of the synthesized samples and to identify the chemical bonds, *ex situ* XPS analyses were performed. The C 1s region shown in Fig. 3 a) is representative of graphene sheets with very low relative amount of oxygen and low nitrogen doping levels: here, for the sample obtained with ammonia as a precursor, the main contributions (> 97 %) to the C 1s profile are the sp^2^ C-C and C-H and the corresponding vibronic fine structure which causes the peak asymmetry. The oxidized species are residual. The tail extends to the high binding energy side due to the energy losses to π-π* excitations (π-plasmons) detected. The XPS quantitative analysis shows a relative atomic concentration of ***nitrogen of 1.5 %***, and a small relative amount of ***oxygen (0.8 %)***. The dominant contributions to the N 1s region (Fig. 3 b) are the peak centred at 399.2 eV, corresponding to ***pyridinic nitrogen (with a fraction of the total N 1s area of 0.66)*** and the peak centred at 400.8 eV from ***pyrrolic nitrogen (whether involved in hydrogen bonding or in an electronegative neighbourhood) and, most probably, quaternary nitrogen (with a fraction of 0.23)***^6^. The feature centred at 403.4 eV is assignable to nitrogen in nitroso groups (-N=O). The computed D parameter (21.5 eV) reflects the strong electron delocalization of the graphene network (Fig. 6 c)^7^.


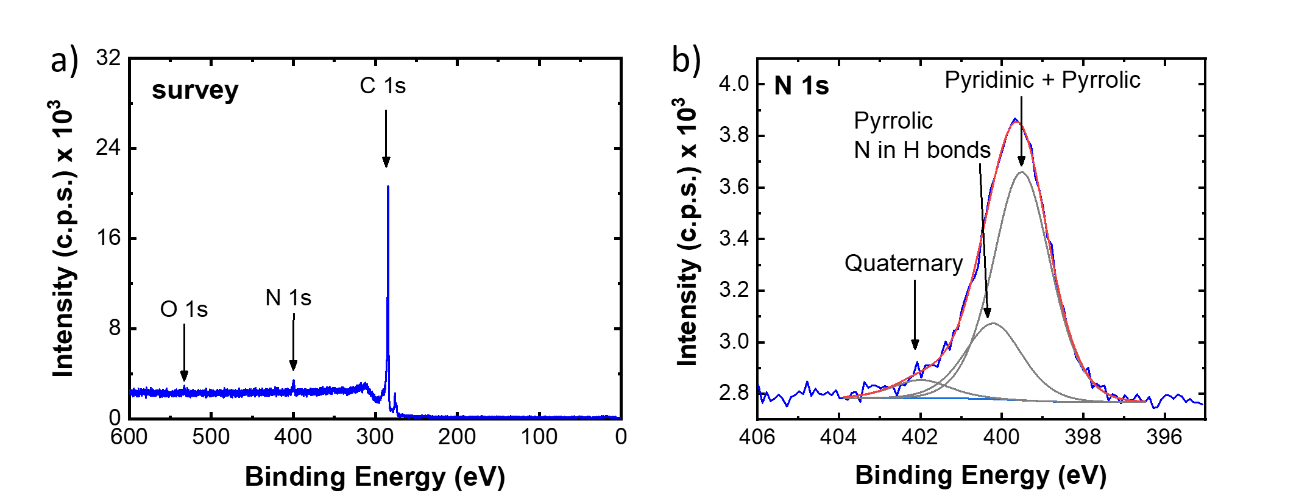


**Figure 4.** (a) Survey XPS spectrum; (b) XPS N1s region, of sample synthesized with methylamine (Q_Ar_ = 1200 sccm, Q_Eth_ = 35 sccm, Q_Meth_ = 3 sccm) in down-to-top scheme.

The use of methylamine as the nitrogen precursor results in a higher doping level: the N 1s region (Fig. 4 a) was fitted with three peaks, centred at 399.5 eV, 400.2 eV and 402.0 eV and assigned to ***pyridinic+pyrrolic nitrogen atoms, pyrrolic nitrogen involved in H bonding and quaternary nitrogen****,* respectively. A ***relatively high nitrogen doping level (Atomic Concentration = 4.4 %) was achieved, with a low oxygen impurity (Atomic Concentration = 1.4 %).*** The survey XPS spectrum (Fig. 4 b) shows the C 1s, O 1s, and N 1s photoelectron peaks.

Furthermore, the nature of chemical intra molecular bonding has been further analysed also by means of NEXAFS. The NEXAFS spectra, presented in Figure (5 a, b) were obtained on the C K-edge and N K-edge, in the partial electron yield mode (PEY), showing the very surface of the substrate. The N-graphene powder was mechanically smeared onto Si wafer. A characteristic sharp C 1s → π* resonance is observable at ~ 285.1 eV (Fig 5 a). The σ* resonances are observed at around 292 eV, with a sharp excitonic peak at ~ 291.7 eV, where π* and σ* refer to anti-bonding molecular orbitals, i.e. bands, of π and σ symmetry, respectively^8^. Higher energy features are due to transitions towards higher-lying states of π or σ symmetry. Features, observed in the region 286 - 290 eV, are typically attributed to the presence of impurities in the graphene lattice. A residual peak observed at $\sim$286.8 eV is attributed to C=N bond. Being nearly one, the intensity ratio of π* and σ* resonances indicates that π electrons conjugated system is well preserved. Furthermore, N K-edge NEXAFS spectra of N-graphene (Fig. 5 b) shows 3 resonance peaks located at $\sim$397.7, 399.7 and $\sim$401.8 eV (signed with A, B,C), which are frequently attributed to pyridinic, pyrrolic and quaternary nitrogen species, respectively, reinforcing the XPS results. Contribution (D) is generally assigned to N 1s core level to C–N σ* states.


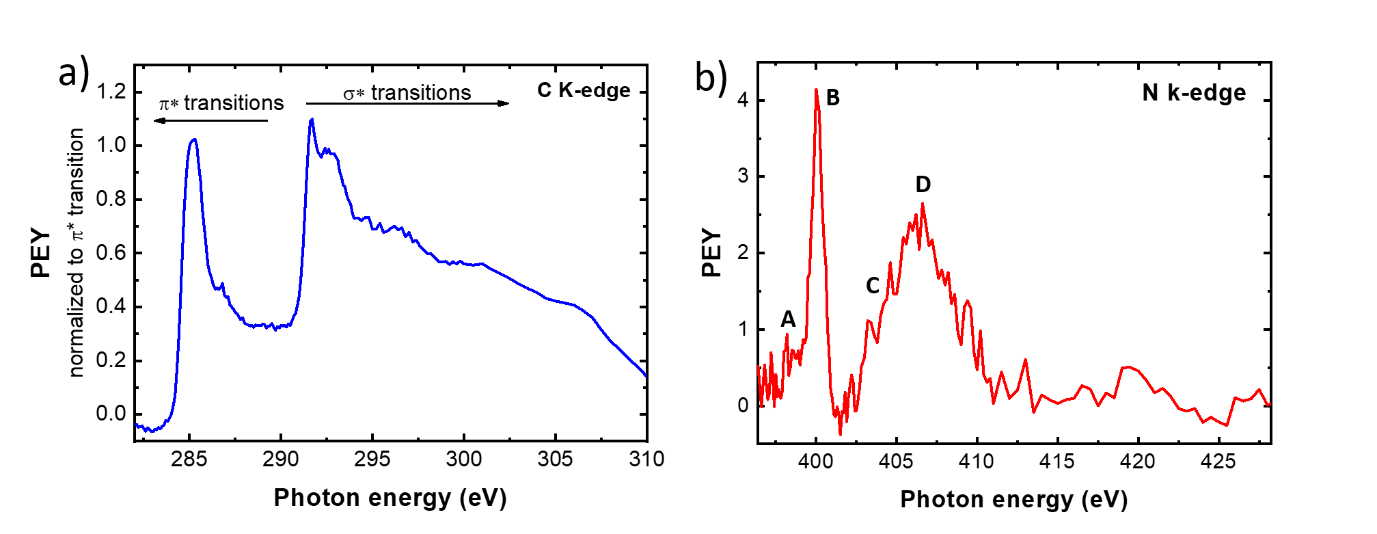


**Figure 5.** (a) C K-edge NEXAFS spectrum; b) N K-edge spectrum of sample synthesized with methylamine precursor (Q_Ar_ = 1200 sccm, Q_Eth_ = 35 sccm, Q_Meth_ = 3 sccm) in down-to-top scheme. C K-edge normalized to the absorption jump, with post-edge intensity at 330 eV set to 1. Both spectra were divided by a clean, freshly sputtered gold sample. Alignment of the energy scale was accomplished by using an I0 feature referenced to a C 1s → π* resonance at 284.9 eV from a fresh surface of a graphite foil standard sample.

To obtain further insight into structural characteristics of the synthesized N-graphene sheets, XRD analysis was performed. Figure 6 shows the obtained XRD patterns of the above considered N-graphene samples. A small shift in the d_002_ peak toward lower angles, corresponding to a very small change of the interlayer spacing, from 3.46 to 3.45 Å is observed for the N-graphene with higher level of doping. However, the full width at half maximum of this peak in the XRD spectrum indicates high level of disorder for sheets with lower level of doping, while at higher doping level the shape indicates more “well organized crystallographic structures”. This demonstrates the change of interlayer spacing that decreases when N doping increases.


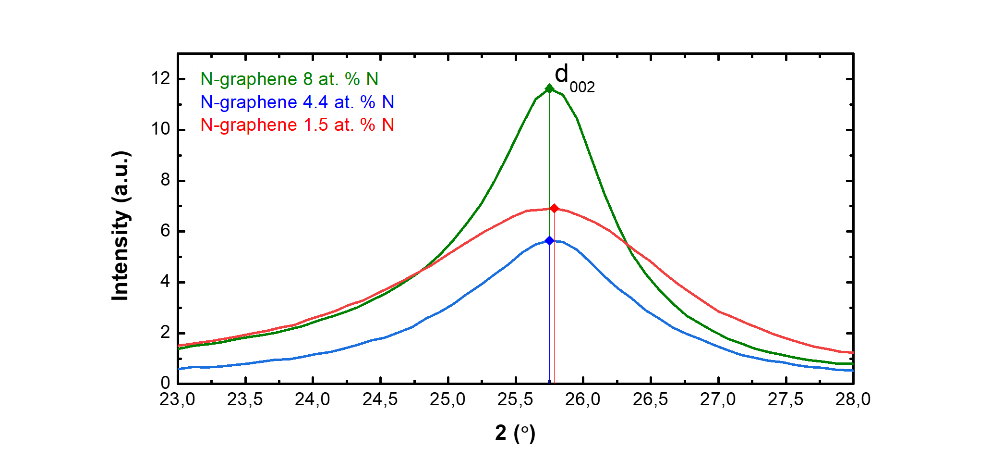


***Figure 6****. X-ray diffraction spectra of N-graphene sheets with different levels of N doping.*

**References**

# 1. Moisan, M., Zakrzewski, Z. Plasma sources based on the propagation of electromagnetic surface waves*. J Phys D: Appl. Phys*. 24 1025 (1991).

2. Tatarova, E. *et al.* Towards large-scale in free-standing graphene/N-graphene sheets. *Scientific Reports* **7**, 10175 (2017), doi:10.1038/s41598-017-10810-3.

3. [Sze, S. M.](https://en.wikipedia.org/wiki/Simon_Sze), Lee, M.-K. Semiconductor Devices: Physics and Technology. New York: Wiley (2016).

4. Ferrari, A. C. *et al*. Raman Spectrum of Graphene and Graphene Layers*. Phys. Rev. Lett.* **97**, 187401 (2006).

## 5. Wu, J.-B., Lin, M.-L., Cong, X., Liua, H.-N. & Tan, P.-H. Raman spectroscopy of graphene-based materials and its applications in related devices. *Chem. Soc. Rev.* 47, 1822-1873 (2018).

6. Gomes, P.J., Ferraria, A.M., Botelho do Rego, A.M., Hoffmann, S.V., Ribeiro, P.A. & Raposo, M. Energy Thresholds of DNA Damage Induced by UV Radiation: an XPS Study. *J. Phys. Chem. B* **119**, 17, 5404-5411 (2015).

7. Kaciulis, S., Mezzi, A., Calvani, P. & Trucchi, D. M. Electron Spectroscopy of the main allotropes of carbon. *Surf. Interface Anal.* **46**, 966-969 (2014).

8. Ehlert, C., Unger, W. E. S. & Saalfrank, P. C. K-edge NEXAFS spectra of graphene with physical and chemical defects: a study based on density functional theory. *Phys. Chem. Chem. Phys.* **16**, 14083-14095 (2014).
